# Supplementary material for: Characterization of microbiota and histology of cultured sea cucumber Isostichopus badionotus juveniles during an outbreak of skin ulceration syndrome
Source: PLoS One. 2024 May 31;19(5):e0303480. doi: 10.1371/journal.pone.0303480 (PMC11142524; doi:10.1371/journal.pone.0303480)
Supplement: Attachment — Submitted filename: Responses to reviewers.docx [file pone.0303480.s003.docx]

**Rebuttal letter**

**Associate Editor**

1. Authors are requested to perform ASV level analyses to mine SUS associated bacterial species.

We recognize the importance of identifying bacterial species associated with SUS. Therefore, we have incorporated an analysis of ASVs at the species level into the paper, complemented by a supplementary table. This section has been entitled “Bacterial species associated with SUS in sea cucumbers” (see lines 344-363).

Moreover, we included a paragraph about this information in the discussion section (see lines 477-487).

2. Authors are requested to describe association of eukaryotic microbes to SUS.

We recognize the importance of characterizing microeukaryotic communities associated with the SUS. However, we have encountered limitations in identifying these communities during an additional analysis that we previously conducted. In that exercise, we analyzed the eukaryotic communities in auricularia larvae of *Isostichopus badionotus* based on 18S rRNA sequencing, however we found that more than 94% of the sequences were taxonomically assigned to the sea cucumber. Consequently, we recognize the need for the development and implementation of blocking primers that inhibit the amplification of *I. badionotus* DNA to achieve a broader representation of these communities in the samples. As a result of these considerations, the present work focused exclusively on the characterization of bacterial communities.

3. Authors are requested to use Italic for all taxonomic names even in higher taxa.

Changes were made to all taxonomic groups as requested.

**Reviewer 1**

1. While the study compares microbiotas in sea cucumbers affected by Sea Cucumber Skin Ulceration Syndrome (SUS), it would benefit from a control group and a larger sample size to strengthen its analysis. Additionally, the novelty of this study should be clarified, as SUS-related microbial changes have been extensively studied before.

We recognize the importance of having a control group and a larger sample size. Initially, we selected two juveniles as control group because they appeared to be healthy. However, upon histological analysis, these juveniles, despite showing no visible skin lesions, exhibited tissue damage similar to that observed in the histological samples of the ulcerated juveniles (see lines 112 to 114). Consequently, we inferred that they might be in the early stage of SUS disease, which could explain the absence of visible lesions at that point. We did not have access to more juvenile sea cucumbers, due to high mortality of these organisms. By this reason, it was not possible to include a control group.

Regarding sample size, the samples used in this study came from the latest group of juveniles obtained under culture conditions in our facilities. At that point, the culture of *I. badionotus* had very limited number of specimens, and its population was significantly reduced due to the occurrence of SUS disease, which initially constrained our sample size. To date, the progress of our experimental culture has been negatively affected by health issues of the early life stages of sea cucumbers. These challenges have prevented us from acquiring new juveniles, thus limiting our ability to expand the sample size or acquire new specimens that could be used as a control group. We highlight the importance of these samples in lines 105-106.

While we acknowledge the extensive study of SUS disease, the novelty of our research lies in introducing, for the first time, the alterations that occur in the bacterial communities associated with SUS in the cultured sea cucumber species *I. badionotus*.

2. Line 88, can you provide the details of filtered seawater? (Nature or artificial, sterilized or not)

This information was included in lines 88-92. “filled with natural seawater obtained from a 24-m deep beach well. The seawater was filtered using a sand filter and treated with ultraviolet (UV) radiation. Furthermore, the sand substrate at the bottom of the containers was also subjected to UV radiation treatment. The tanks were part of a closed recirculating system formed by a sand filter, a biological filter, continuous UV radiation (2 x 40 W), and a water chiller fixed at 25 °C.”

3. Line 90, I didn't quite understand, the water was renewed at 60% of new water?

This information was clarified in line 93-94. “To remove faces and unconsumed food, 60% of the seawater was replaced every two days”.

4. Line 108, sample size might be too small, I think it should be at least 3 samples per group.

We recognize that sample size was small, but it was due to the reasons mentioned in question 1 above. Nevertheless, additional sample replications were conducted to address the limited sample size and enhance the robustness of the study (see lines 125-130).

5. Line 109, no control/ healthy group?

This information was addressed in the first question above.

6. Line 119, I am wondering the stage of SUS of these four juveniles, you mentioned about two of them were healthy when you collecting the samples, how about the others? If they were in disease, how could you collected the diseased microbiota after washing the surfaces?

The information about the remaining two juveniles was clarified in line 114-116. “The other two juveniles exhibited signs of disease, manifested by visible ulcers on their skin, and were classified as being in the advanced stage of the disease”.

The surface washing was conducted to eliminate bacterial communities not specific to the skin sample, which could introduce interference as potential contamination. This contamination might arise from water and organic matter accumulated in the culture tanks (refer to line 123). This methodology has been previously presented and discussed by Tangestani and Kunzmann, 2019 (reference 15). The ulcers observed on the skin of our juveniles extended down to the dermis, making it unlikely that the communities present at that level were affected by the superficial washings. Moreover, these washes were essential to prevent contamination of internal organs during the dissection of the organisms.

7. Line 125, I didn't understand the " three from the second one", do you mean the stage between early to advanced?

We rewrite this paragraph to make easier to understand the ideas. See lines 127-130, “For molecular analysis, we collected eight skin samples from juveniles in the early stage of SUS (four samples from each juvenile). Additionally, seven skin ulcer samples were collected from juveniles in the advanced stage of SUS (three ulcer samples from one organism and four from the other one)”.

8. Line 260, since you compared microbiotas between different sections and individuals, how do you know the differences were caused by disease or individual variation?

While we physically separated the anterior and posterior parts of the gut during sampling, we did not conduct a specific analysis of the microbiota between these gut sections. Instead, the analysis considered both sections as a whole. We clarified this information in the lines 176-178. This approach aimed to represent the overall bacterial diversity of the sea cucumber digestive tract to facilitate a comparison between the early and advanced stages of SUS disease). Unfortunately, as mentioned above, it was not possible to increase the sample size for this study.

9. Line 391, it is not a complete sentence.

Thank you for your comment, this information was not informative, and it was deleted. Additionally, we clarified the information in line 421-422. “This necrosis is characterized by dead tissue's partial or complete dissolution resulted from infection with microbial agents (bacteria, fungi, viruses, and parasites).

10. Line 419-426, since you mentioned about the differences of microbiome caused by culture condition and section of intestine, how could you know the change of specific bacteria is associated with the SUS?

The information was clarified in line 445-453. “This similarity may be attributed to the observation that the bacterial species composition in the early stages or initial hours of SUS disease development is the same as that of healthy organisms [21,36]. This similarity may also be related to the time that sea cucumbers have spent in captivity (six months), since the composition of the intestinal microbiota is influenced by both intrinsic factors (genetics, immune status, life stage) and extrinsic factors (diet and environmental conditions) [45-47]. The same pattern has been observed in *Holothuria glaberrima*, where significant changes in the intestinal microbiota were observed between wild cucumbers and those maintained in aquaria for only three days [45]”.

We cannot definitively establish a link between the specific bacterial changes observed in our study and the SUS disease, primarily due to the absence of a control group and the lack of microbiota analysis by intestinal sections. Nevertheless, it is apparent that the microbiota exhibited significant differences between the two presented juvenile categories. Moreover, the outcomes of histological analyses indicating variations in the severity of observed pathologies, provided compelling evidence to suggest that changes in the bacterial communities are highly likely associated with the progression of the disease. For this reason, we have decided to remove the text on line 453: “Moreover, differences can also be influenced by the specific section of the intestine, its anatomical composition and its function [12]”, to avoid confusion.

11. Line 454, can you explain why *Vibrio* decreased in advanced stage of SUS in your study?

A discussion of the possible reasons for the decrease in *Vibrio* abundance in our study has been added in the lines 494-496. “Different species of *Vibrio* can be of benefit for animals, and not only pathogens. Actually, in aquaculture there are examples of the use of *Vibrio* strains as probiotics [52–54]. Therefore, our results suggest that a decrease of the abundance of *Vibrio* during SUS-disease progression could be related to a reduction of beneficial *Vibrio* strains in the intestine of *I. badionotus* juveniles, due to stress conditions into the host”.

12. Line 499, could you add some discussion about how can you prevent the spread of the disease according to your result?

The discussion was added in lines 541-546. “Therefore, the implementation of longitudinal monitoring of microbial communities in culture systems, including those in the seawater used for culture, is necessary to implement timely interventions, such as adjustment of culture conditions or administration of probiotics. This is especially important in the case of bacterial genera reduced during disease progression, which in turn could prevent the spread of SUS in the *I. badionotus* culture.”
