# Supplementary material for: Characterization of microbiota and histology of cultured sea cucumber Isostichopus badionotus juveniles during an outbreak of skin ulceration syndrome
Source: PLoS One. 2024 May 31;19(5):e0303480. doi: 10.1371/journal.pone.0303480 (PMC11142524; doi:10.1371/journal.pone.0303480)
Supplement: Attachment — Submitted filename: Responses to Reviewers.docx [file pone.0303480.s004.docx]

**Rebuttal letter**

**Reviewer 1**

1. I understand the difficulty in collecting the control groups. However, I suggest adding a discussion about the microbiota of healthy *I. badionotus*. This addition would help clarify the differences in bacterial groups between diseased and healthy *I. badionotus* and provide insight into which bacteria may be inducing the disease.

We recognize the importance of adding information about the microbiota of healthy *I. badionotus*. However, we consider that even with this additional information, the findings of our study do not allow us conclusively to determine which bacteria induce the disease. This is because, as mentioned in lines 58-60, this disease is multifactorial and can be caused by biotic and abiotic factors, or a combination of both. Infection assays, such as those performed by Delroisse et al. (2020), are needed to confirm whether the proposed bacteria cause the disease.

Only two studies have reported the microbiota composition of healthy *I. badionotus* in the scientific literature. The first, by Luna-Fontalvo et al. (2014), focused on characterizing the diversity of culturable bacteria from the skin and gut of *I. badionotus* collected in Colombia. However, this study does not present the role of these organisms in sea cucumbers and does not provide information on the relationship between these bacteria and the disease.

The second study by Quintanilla-Mena et al. (2022) used 16s rRNA gene sequencing to characterize the microbiota in different sections of the digestive tract of *I. badionotus* maintained in captivity for six months in Telchac, Yucatan. This study shares methodological and geographic similarities with our research. However, their samples were collected from sea cucumbers maintained in captivity for a period of time, while ours are from fully cultured sea cucumbers. Due to these differences in the origin of the samples, differences in the microbiota composition are likely to be observed. Nevertheless, we decided to base our discussion of the microbiota of healthy *I. badionotus* on their results, since the study of Quintanilla-Mena et al. (2022) provides the closest comparison to the microbiota of a healthy *I. badionotus*. Furthermore, we extended the discussion to the genus level to better identify possible changes or similarities in gut microbiota composition (see lines 436-459).

“The intestinal microbiota composition of our early SUS *I. badionotus* juveniles was mainly composed of *Proteobacteria* (50.2%), *Bacteroidetes* (25.7%), *Campylobacterota* (12.3%) and *Actinobacteria* (3.1%), with *Firmicutes* present at a lower relative abundance of 2.8%. These results were similar at the Phylum level to those found in sea cucumbers of the same species kept in captivity (organisms collected from wild populations and kept in fiberglass tanks for six months) [12]. These authors reported that *Proteobacteria* (60.9%), *Actinobacteria* (11.6%), and *Bacteroidetes* (6.8%) were the predominant bacterial Phyla in the intestine. Nevertheless, the relative abundance of *Firmicutes* (12.9%) was higher than our results.

At the genus level, high abundances of *Vibrio* (10.2%), as well as the presence of *Shimia* (3.7%), *Halarcobacter* (syn. *Arcobacter*) (3.8%), and *Thalassotalea* (2.5%), were observed in the intestine of early SUS juveniles. These results were similar to those observed in the hindgut of captive *I. badionotus*, where *Vibrio* (17.4%) and *Shimia* (11.7%) were the most abundant after *Ruegeria*. The low relative abundances of *Arcobacter* and *Thalassotalea* in the present study may be similar to those found in the captive sea cucumbers. This similarity may be attributed to the observation that the composition of bacterial species in the early stages of the initial hours of SUS disease development is the same as that of healthy organisms [21,36]. This similarity may be attributed to the observation that the composition of bacterial species in the early stages of the initial hours of SUS disease development is the same as that of healthy organisms [21,36]. This similarity also may be related to the time that sea cucumbers have spent in captivity (six months), which can influence the composition of the intestinal microbiota. The gut microbiota of sea cucumbers is influenced by both intrinsic factors such as genetics, immune status, and life stage, and extrinsic factors such as diet and environmental conditions [45–47]. Significant changes in the gut microbiota have been observed in *Holothuria glaberrima* between wild sea cucumbers and those maintained in aquaria for just three days [45]. *Vibrio* is a genus that has been found in high abundance in the intestines of healthy *A. japonicus* and *H. glaberrima* [45,48–50]. Therefore, this genus appears to be part of the normal microbiota of sea cucumbers.”

2. Regarding the ASV analysis, could you consider conducting a more in-depth analysis? For example, you could focus on significantly changed ASVs and the most abundant ASVs at specific stages.

We agree that focusing on the ASVs that have undergone significant changes at each disease stage is important. In fact, the LEFSE analysis was performed to compare the bacterial genera producing changes in the composition and structure of the microbiota of cultured *I. badionotus* at both disease stages (early and late).

In this analysis, we observed that *Vibrio* and *Thalassotalea* are two relevant genera in the early stage of SUS, both in the skin and intestinal microbiota. However, the identification of *Vibrio* in the gut microbiota of *I. badionotus* supports the association of this genus with the early stage of the disease.

On the other hand, *Halarcobacter* (syn. *Arcobacter*) represents the bacterial genus with the highest significant association in both the skin and intestine of juvenile *I. badionotus* during the advanced stage of SUS. However, as we indicated in lines 38-39, this is not the only bacterial genus that could be involved in the development of the disease, since 15 other genera were identified as contributing to the change in the composition and structure of the microbiota during this stage. Nevertheless, the increase in the abundance of *Halarcobacter* (syn. *Arcobacter*) from the early to the late stage of SUS suggests that they play an important role in developing the disease.

3. I still have some confusion regarding the role of *Vibrio* in this context. Have any reference studies shown a decrease in *Vibrio* during the SUS? Clarifying this point would strengthen the discussion surrounding *Vibrio*'s involvement in the disease process.

To answer this question, we added the following text in lines 520-535.

“Currently, no scientific evidence indicating a decrease in the *Vibrio* bacterial genus during SUS. *Vibrio* is a bacterium typically found in sea cucumber species such as *I. badionotus*, *A. japonicus*, and *H. glaberrima*, and is known to be beneficial for them. However, a decrease in the abundance of *Vibrio* can have significant consequences because it allows for the colonization and proliferation of potentially pathogenic and opportunistic bacteria, such as *Halarcobacter* (syn. *Arcobacter*), that has also been identified as part of the microbiota of healthy sea cucumber organisms [12,58].

A similar phenomenon has been observed in the gut microbiota of juvenile *A. japonicus*, where the antibiotics significantly decreased the abundance of beneficial bacteria such as *Vibrio* and *Thalassotalea*, which were dominant bacterial genera in their microbiota along with *Halarcobacter* (syn. *Arcobacter*). However, the decrease in the abundance of these bacterial genera led to a significant increase in the abundance of *Halarcobacter* (syn. *Arcobacter*), which increases the risk of host infection [58]. Furthermore, an increase in the abundance of *Arcobacter* spp. has been reported in both internal and exposed tissues of diseased or stressed fish, oysters, and shrimp [63], suggesting this bacterium´s pathogenic potential due to microbiota alterations.”
